# A Concordance Between Clinical and Pathological Tumor Staging of Oral Squamous Cell Carcinoma: An Institutional Study

**DOI:** 10.7759/cureus.61584

**Published:** 2024-06-03

**Authors:** Dharini S, Karthikeyan Ramalingam, Pratibha Ramani, Murugesan Krishnan

**Affiliations:** 1 Oral Pathology and Microbiology, Saveetha Dental College and Hospitals, Saveetha Institute of Medical and Technical Sciences, Saveetha University, Chennai, IND; 2 Oral and Maxillofacial Surgery, Saveetha Dental College and Hospitals, Saveetha Institute of Medical and Technical Sciences, Saveetha University, Chennai, IND

**Keywords:** tnm, tumor size, tumor staging, tumor grade, computed tomography, quality of life, prognostic indicator, oral cancer, nodal metastasis, clinical examination

## Abstract

Background: Among oral diseases, oral cancer is the primary cause of death and poses a serious health risk. Primary tumor (T) - regional lymph node (N) - distant metastasis (M) comprising (TNM) staging is crucial for planning treatment strategies for patients with oral squamous cell carcinoma (OSCC).

Aim: This study evaluated the predictive accuracy of clinical TNM staging of OSCC to histopathological staging (pTNM) in an institutional setting.

Materials and methods: Fifty-four consecutive histologically confirmed, surgically treated OSCC cases were evaluated for TNM staging. The study compared the clinical staging at the time of surgery with the pathological staging obtained from excisional biopsy reports. Microsoft Excel (Microsoft® Corp., Redmond, WA, USA) was used for the data compilation and descriptive analysis. The chi-square test, analysis of variance (ANOVA), and Tukey's Honest Significant Difference (HSD) posthoc test were used to compare the data for statistical significance with p value <0.05 using Statistical Package for the Social Sciences (IBM SPSS Statistics for Windows, IBM Corp., Version 23.0, Armonk, NY).

Results: The alveolar mucosa (n=22, 40.74%) was the most frequently occurring site, followed by the tongue (n=17, 31.48%). Out of the 54 included cases, based on clinical tumor size, there were T1 (n=6), T2 (n=13), T3 (n=13), T4a (n=16) and T4b (n=6). T2 tumors were usually upstaged (n=7) while T4a (n=8) tumors were most often downstaged. T4a (n=8) had the best concordance between clinical and histopathological staging, followed by T2, T3, and T1. In nodal status, N1 showed the most variation. The chi-squared test showed statistical significance for tumor size comparison (p <0.001) and nodal status comparison (p=0.002). ANOVA test did not show any statistical significance. Tukey's HSD posthoc test showed statistical significance (p=0.034) for N0 and N1 status. The highest concordance was shown by N0 and N1 followed by N2b.

Conclusion: Preoperative radiological and clinical assessments are essential for deciding on a patient's course of treatment. However, not all patients may require radiographs to determine tumor size or nodal status assessment. Accurate diagnosis is vital for the treatment planning of OSCC.

## Introduction

In many countries, oral cancer is the primary cause of death among oral diseases and it presents a significant health risk. The year 2018 had 177,384 deaths and 354,864 new cases according to recent global estimates. Traditional risk factors such as alcohol and tobacco use contribute to the initiation of oral cancer [1]. Oral squamous cell carcinoma (OSCC) is the most prevalent head and neck malignancy, and it is becoming more common worldwide, particularly in young adults [2]. It's still up for debate whether people with OSCC who are younger or older have different outcomes [3,4].

Nevertheless, no appreciable differences in tumor staging or histopathological grading were found in recent investigations comparing the features of OSCC in different age groups [5]. In comparison to other subsites like the floor of the mouth, gingiva, and retromolar trigone, OSCC is more common in the tongue, and its associated mortality is higher, according to a recent analysis of SEER (Surveillance, Epidemiology, and End Results) [6]. Surgical resection is the mainstay of treatment for OSCC; it can be used either in isolation or in conjunction with adjuvant therapies like radiation or chemoradiotherapy. Several factors, including differentiation, growth pattern, depth of invasion (DOI), margin status, presence of vascular/neural invasion, bone involvement, nodal status (number of lymph nodes involved, size of largest metastasis, presence of extracapsular spread/extranodal extension), and pTNM staging, are taken into consideration when deciding whether to administer adjuvant treatment for a resected tumor. A framework for determining prognosis and developing stage-specific, guideline-driven treatment has been made available by cancer staging. A recent international version released in the United Kingdom in 2005 by the Royal College of Pathologists added the concept of standard histopathology reporting [7].

The TNM (tumor, node, metastasis) staging system is principally used by the American Joint Committee on Cancer (AJCC) to characterize and stage tumor extension. Additionally, this system directs therapeutic approaches according to internationally accepted standards. It is also essential for treatment planning, recurrence risk estimation, and overall survival assessment of the patients [8,9]. Studies assessing clinical staging in patients with OSCC have not been carried out frequently among the Indian population. Therefore, the present study was planned to evaluate the predictive value of clinical TNM staging with pathological TNM staging among OSCC patients.

## Materials and methods

The study analyzed biopsy-proven OSCC patients who were treated in our institution. Ethical clearance was obtained from the Institutional Human Ethical Committee vide letter number - (IHEC/SDC/Phd/Opath-1954/19/TH-001). Data collection utilized the Dental Information Archival System (DIAS), an institutional software of Saveetha Dental College and Hospitals, Chennai.

Histologically confirmed OSCC patients who received surgery as primary treatment were included in this study. There were no age or gender restrictions. Patients with other head and neck pathologies like salivary tumors, odontogenic tumors, and soft tissue tumors other than OSCC, patients who underwent neoadjuvant chemo or radiotherapy, and patients with recurrence were excluded.

Demographic and clinical details, including gender, age, clinical TNM at the time of surgery, histopathological data on tumor grading, and pathological TNM (pTNM) staging were collected. Staging was recorded as per the eighth edition of the AJCC for oral cavity carcinoma. T indicates primary tumor, N indicates regional lymph node and M indicates distant metastasis.

Based on the size of the tumor and DOI, T is categorized into Tx (primary tumor cannot be assessed), Tis (carcinoma in-situ), T1 (characterized by size of 2 cm or smaller and a DOI less than 5 mm), T2 (defined either by size of 2 cm or smaller with a DOI greater than 5 mm, or by a size between 2-4 cm with a DOI of ≤10), T3 (shows tumor size either larger than 4 cm with a DOI less than 10 mm, or between 2-4 cm with a DOI greater than 10 mm) and T4 signifies moderately advanced tumors (T4a) to very advanced local disease (T4b). T4a has a tumor size of >4 cm with >10 mm DOI. It shows tumor invasion to adjacent structures such as the cortical bone of the mandible or maxilla, maxillary sinus, or skin of the face whereas T4b tumors encase the internal carotid artery and/or invade the pterygoid plates, skull base, and/or masticator area [7,8].

Clinical N (cN) is classified into cNx (no lymph nodes can be evaluated) and cN0 (no lymph node metastases), cN1 (single lymph node metastasis on the same side, with no extranodal extension (ENE) visible and a maximum size of ≤3 cm or less), cN2 (cN2a, cN2b, cN2c) and cN3 (cN3a, cN3b). cN2 includes metastasis in a single lymph node on the same side, larger than 3 cm but not exceeding 6 cm, without ENE (N2a); metastasis in multiple lymph nodes on the same side, none larger than 6 cm and without ENE (N2b) and metastasis in lymph nodes on both sides or the opposite side, none exceeding 6 cm and without ENE (N2c). N3 denotes metastasis in a lymph node larger than 6 cm without ENE (cN3a) or metastases in any node(s) with clinically apparent ENE (cN3b) [7,8].

Pathological N (pN) classification includes pN0, pN1, pN2 (with subcategories pN2a, pN2b, and pN2c), and pN3 (with subcategories pN3a, pN3b). The categories pN0 and pNx correspond to cN0 and cNx respectively. pN1 is defined by metastasis in a solitary lymph node on the same side, without ENE, and with a maximum diameter of ≤3 cm. pN2 indicates metastasis in one lymph node on the same side that is 3 cm or smaller and has an ENE+ or 3 to 6 cm without ENE- (pN2a); it can also occur in multiple lymph nodes on the same side that are none larger than 6 cm without ENE- (pN2b) and it can also occur in lymph nodes on both sides or the opposite side but nodes are not larger than 6 cm without ENE- (pN2c). pN3 includes pN3a (metastasis in a lymph node larger than 6 cm without ENE-) and pN3b (metastases in a single lymph node on the same side larger than 3 cm with ENE+ or multiple lymph nodes on the same side, opposite side, or both sides with ENE+ or a single lymph node on the opposite side of any size with ENE+. M1 indicates distant metastasis whereas M0 denotes a tumor with no distant metastasis [7,8].

Clinical TNM was categorized into cT1, cT2, cT3, cT4a, and cT4b, and nodal status into cN1, cN0, cNx, cN2a, cN2b, cN2c, cN3a and cN3b. Histopathological TNM of the included cases involved tumor size and lymph node metastasis, comprising cases classified as pT1, pT2, pT3, and pT4a for tumor size, and pN0, pNx, pN1, pN2a, pN2b, pN2c, pN3a and pN3b for nodal status [7,8].

Distant metastasis was not considered in this study, as a full-body scan and biopsy of the identified metastasized site are required to compare the clinical and pathological findings. Clinical staging was compared with pathological staging obtained from an excisional biopsy of oral cancer patients. Data were descriptively analyzed using MS Excel (Microsoft® Corp., Redmond, WA, USA) and statistical analysis was carried out using the Chi-square test, analysis of variance (ANOVA), and Tukey's Honest Significant Difference (HSD) posthoc test (p<0.05) using Statistical Package for the Social Sciences (IBM SPSS Statistics for Windows, IBM Corp., Version 23.0, Armonk, NY).

## Results

According to the study’s inclusion criteria, 54 patients histopathologically diagnosed with OSCC were selected. There were 40 cases (74%) of male and 14 cases (26%) of female patients. The age distribution ranged between 31 and 73 years. The age group most frequently observed in the present study was between 51 and 60 years, accounting for 40.74% of the cases, with 22 patients falling within this age range.

**Figure 1:**
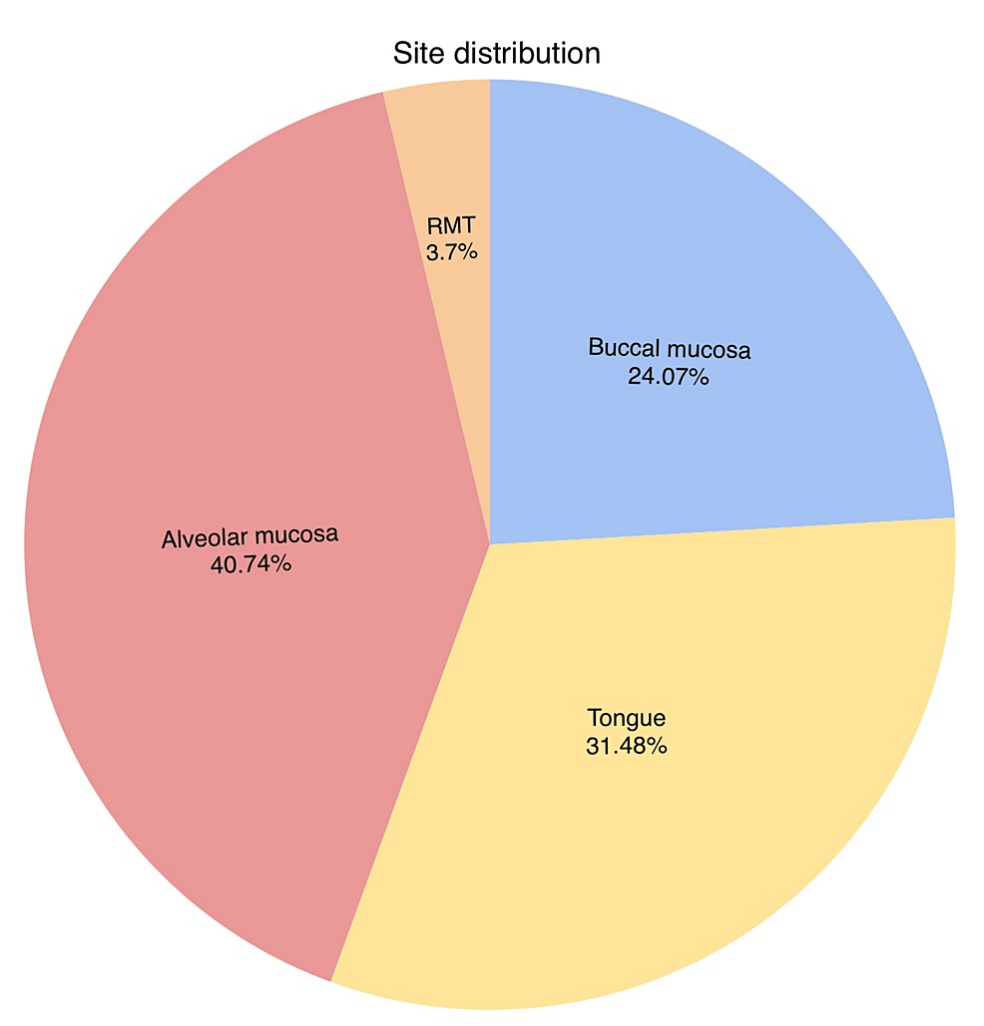
Anatomical site distribution of oral squamous cell carcinoma in our study RMT: retromolar trigone

The most common site of occurrence was 22 in alveolar mucosa (40.74%) followed by 17 in the tongue (31.48%), 13 in buccal mucosa (24.07%), and two in retromolar trigone (3.7%) (Figure 1).

**Figure 2:**
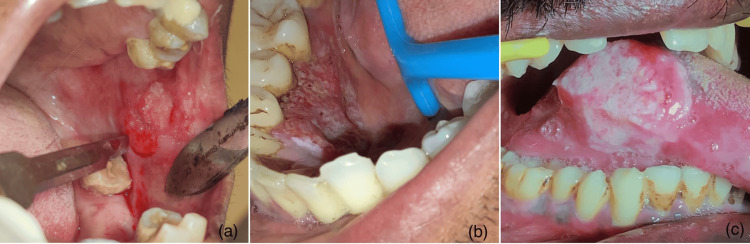
Clinical images of oral squamous cell carcinoma Clinical images showing (a) T1 tumor on the left buccal mucosa, (b) T2 tumor on the right mandibular lingual gingiva and vestibular region, and (c) T3 tumor on the right lateral border of the tongue.

**Figure 3:**
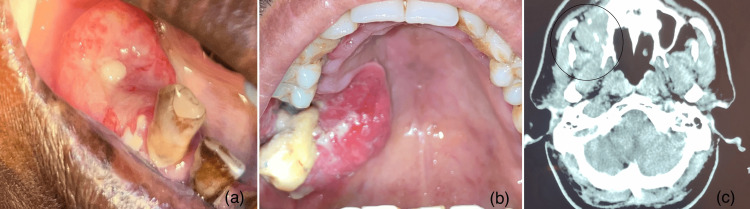
Clinical and radiological images (a) T4a tumor on the right mandibular alveolus; (b) T4b tumor on the right posterior palate; (c) Computed tomography image showing bone destruction concerning right maxilla.

Six cases of T1, six cases of T4b, 13 cases of T2, 13 cases of T3, and 16 cases of T4a out of the 54 patients based on the clinical tumor size (Figures 2, 3).

The 54 cases evaluated in the present study include T1 (n=6, 11.1%), T2 (n=13, 24.07%), T3 (n=13, 24.07%), T4a (n=16, 29.63%) and T4b (n=6, 11.1%) based on tumor size, N0 (n=9, 16.66%), Nx (n=1, 1.85%), N1 (n=33, 61,1%), N2a (n=3, 5.55%), N2b (n=3, 5.55%) and N2c (n=5, 9.26%). The cases were clinically staged as follows: Stage I (n=2, 3.7%), Stage II (n=3, 5.55%), Stage III (n=22, 40.74%), Stage IV A (n=19, 35.19%), Stage IVB (n=6, 11.1%), and Stage IVC (n=2, 3.7%).

**Figure 4:**
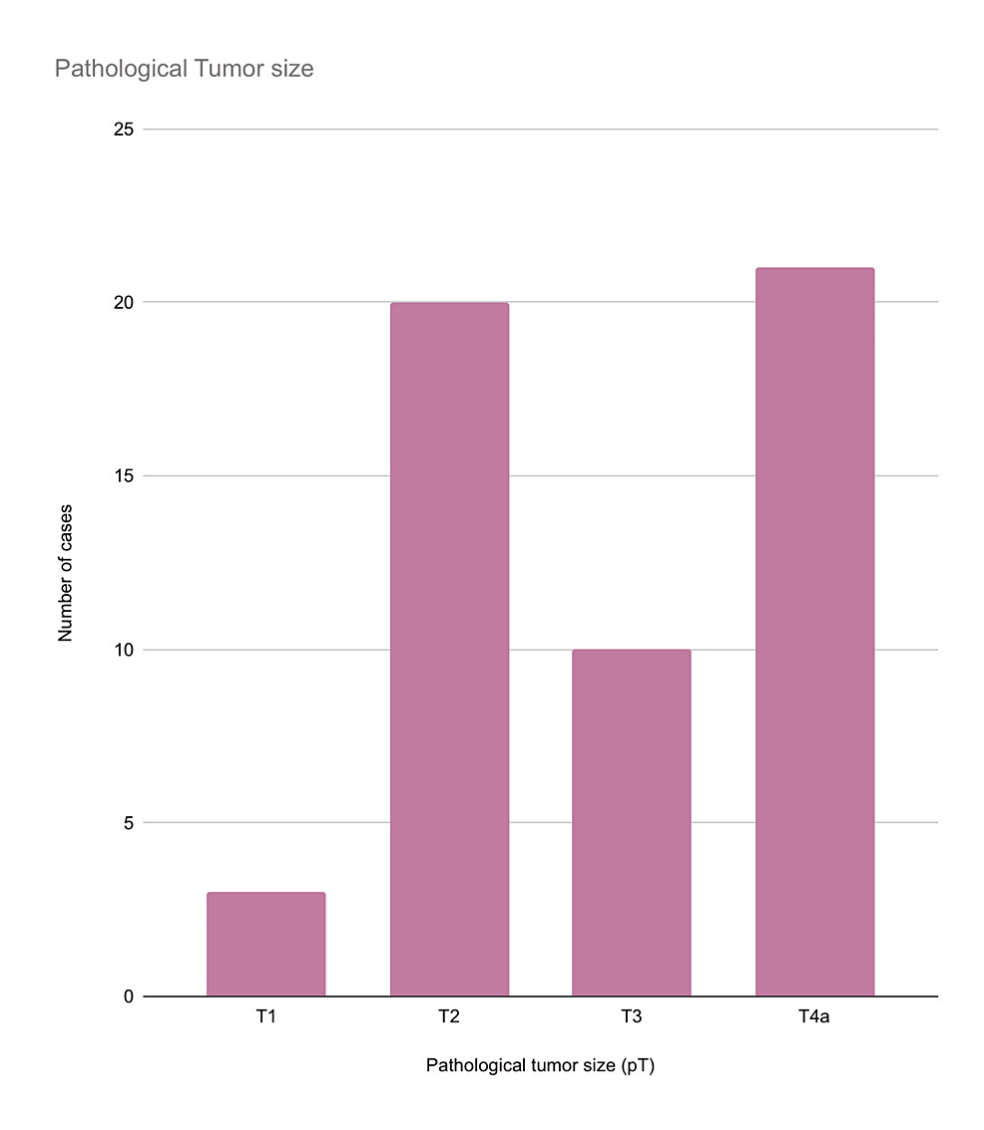
Distribution of pathological tumor size among the included cases

Among the 54 cases, three (5.5%) cases were classified as T1, 20 (37%) cases as T2, 10 (18.52%) cases as T3, and 21 (38.89%) cases as T4a based on the pathological tumor size (Figure 4).

**Figure 5:**
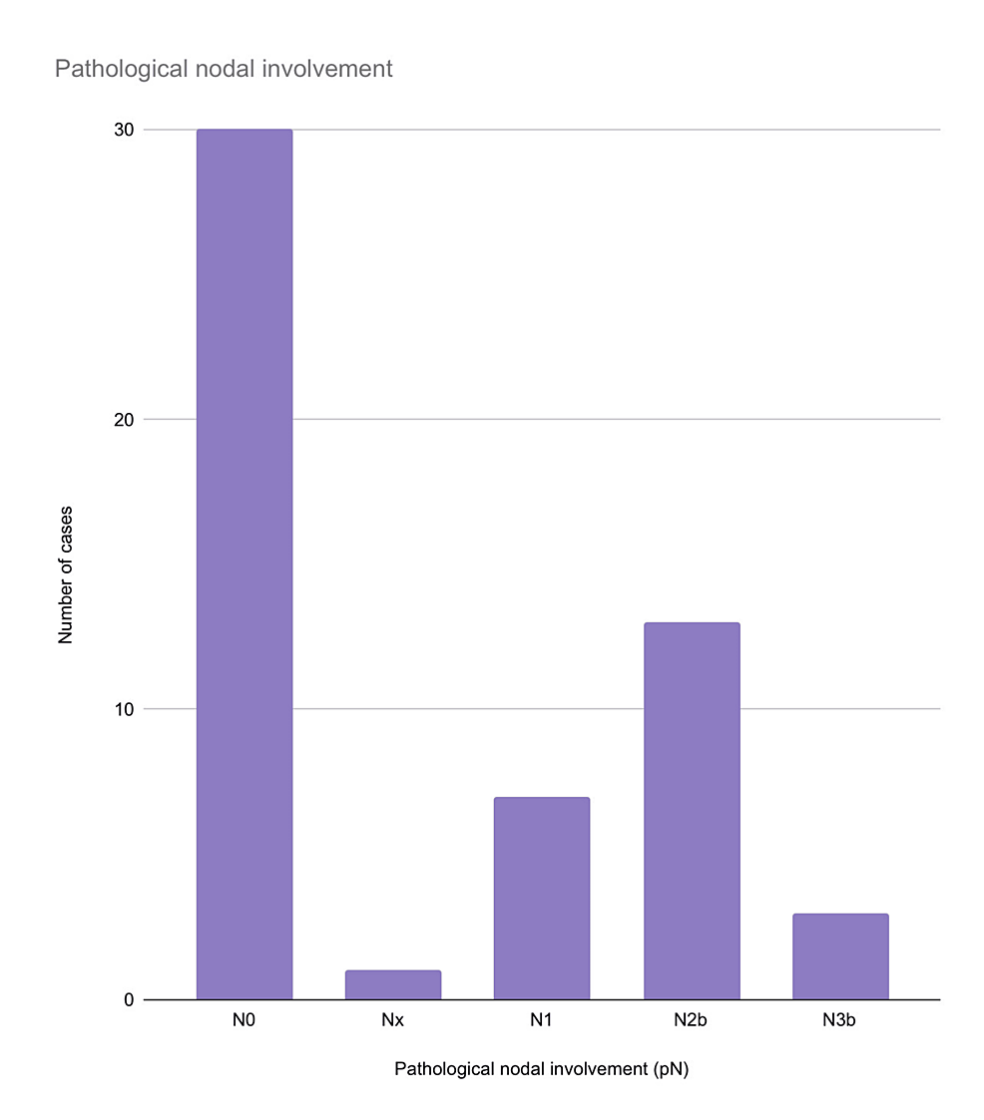
Distribution of pathological nodal status among the included cases

Thirteen (24.07%) patients had N2b nodal status, seven (12.96%) had N1 nodal status, one (1.85%) had Nx nodal status, three (5.56%) had N3b nodal status and 30 (55.55%) had N0 nodal status (Figure 5).

**Figure 6:**
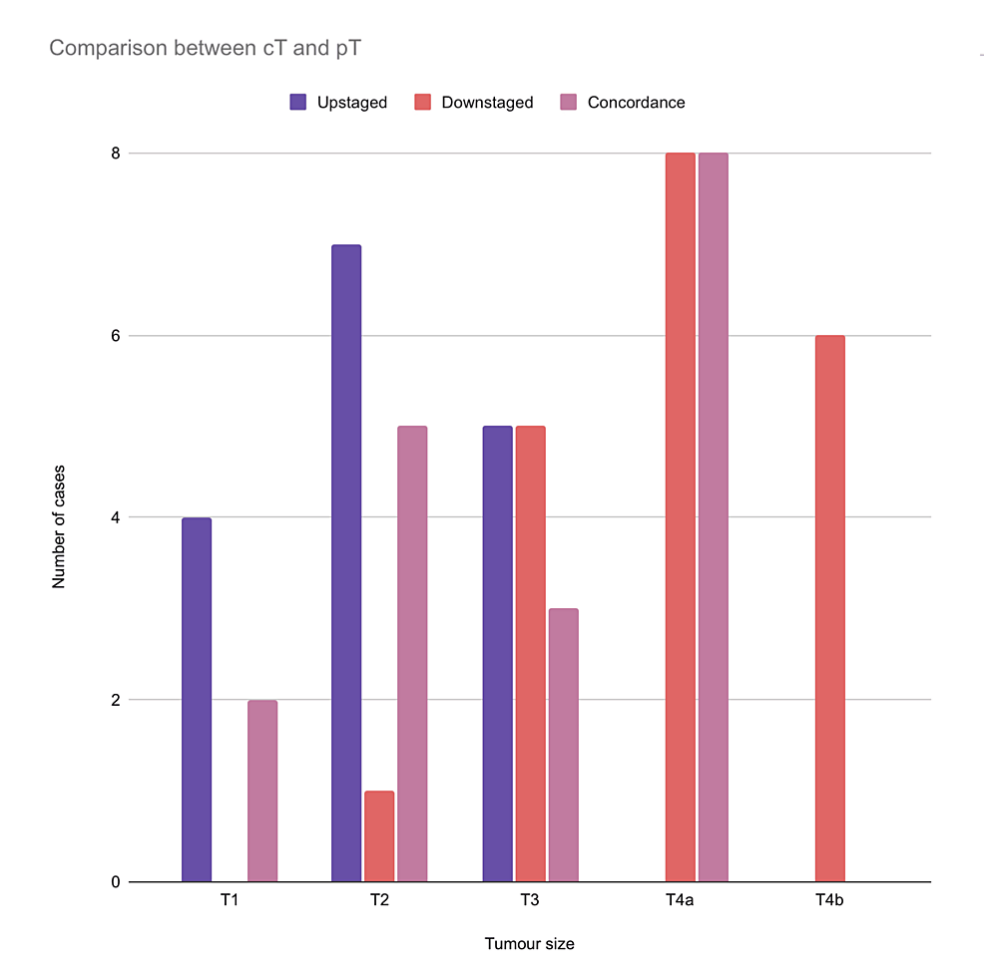
Comparison between clinical tumor size and pathological tumor size among the included cases

T2 (n=7, 12.96%) tumors were commonly upstaged, followed by T3 (n=5, 9.26%) and T1 (n=4, 7.41%). T4a (n=8, 14.81%) and T4b (n=6, 11.1%) tumors were frequently downstaged, followed by T3 (n=5, 9.26%) and T2 (n=1, 1.85%). T4a (n=8, 14.81%) was in concordance in most instances followed by T2 (n=5, 9.26%), T3 (n=3, 5.55%), and T1 (n=2, 3.7%) (Figure 6).

The Chi-square test was employed to compare the overall tumor scoring per clinical and pathological TNM which was statistically significant with a p-value of less than 0.001 for tumor size and less than 0.002 for nodal involvement (Table 1).

**Table 1:** Chi-square test for tumor size and nodal involvement * Statistically significant

Chi-square (Tumor Size)			
	Value	df	Asymp. Sig. (two-sided)
Pearson Chi-Square	28.458	8	<0.001*
Likelihood ratio	37.126	8	<0.001
Linear-by-linear association	3.922	1	0.048
No. of valid cases	54		
Chi-square (Nodal Involvement)			
	Value	df	Asymp. Sig. (two-sided)
Pearson Chi-Square	27.839a	10	0.002*
Likelihood ratio	30.579	10	0.001
Linear-by-linear association	0.362	1	0.547
No. of valid cases	54		

ANOVA test for the tumor size was not statistically significant with a p-value of 0.080. It was not statistically significant for nodal involvement with a p-value of 0.051 (Table 2).

**Table 2:** ANOVA comparison between clinical and pathological tumor size and nodal involvement of the included cases ANOVA: analysis of variance

ANOVA (Tumor Size)					
Overall Outcome					
	Sum of Squares	df	Mean Square	F	Sig.
Between Groups	5.208	4	1.302	2.222	0.080
Within Groups	28.718	49	0.586		
Total	33.926	53			
ANOVA (Nodal Involvement)					
	Sum of Squares	df	Mean Square	F	Sig.
Between Groups	4.383	4	1.096	2.554	0.051
Within Groups	20.598	48	0.429		
Total	24.981	52			

**Figure 7:**
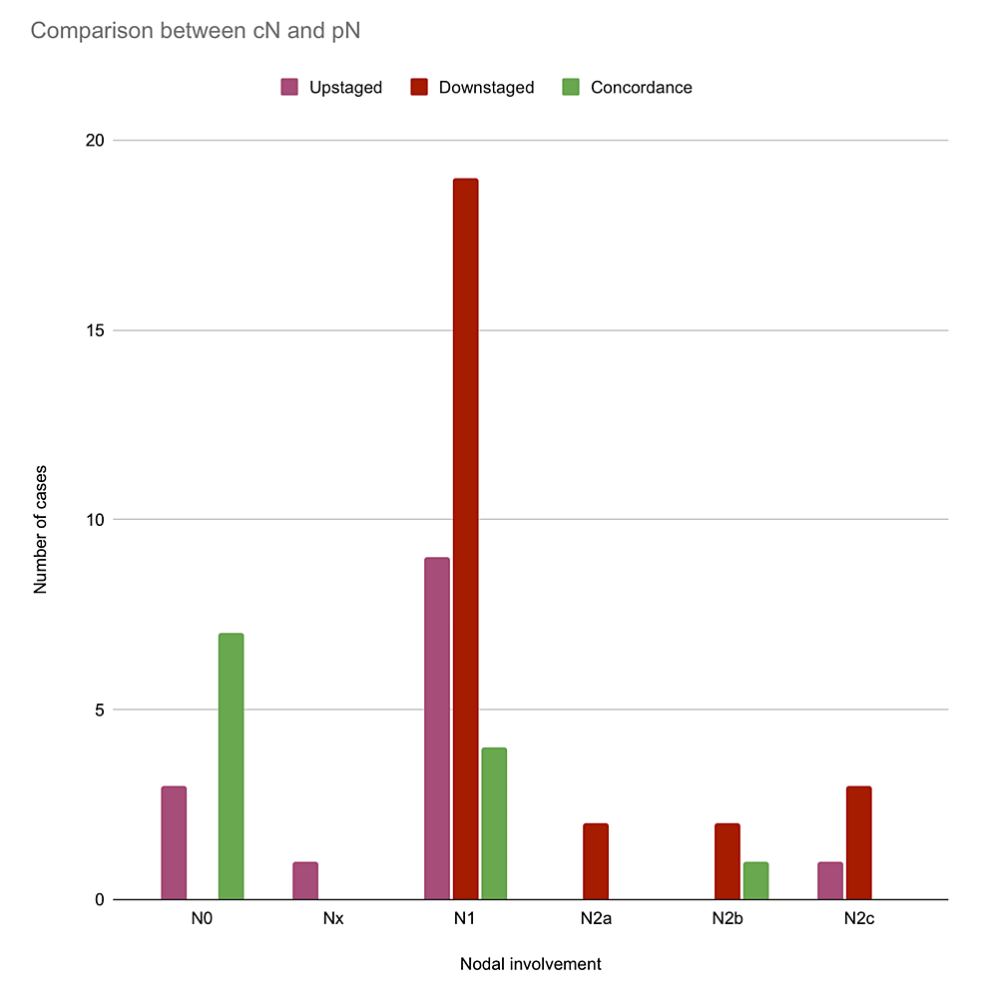
Comparison between clinical nodal status and pathological nodal status among the included cases

Similarly, for nodal status, N1 (n=9) was often upstaged, followed by N0 (n=3), Nx (n=1), and N2c (n=1). Conversely, N1 (n=19) was frequently downstaged, followed by N2c (n=3), N2b (n=2), and N2a (n=2). N0 (n=7) and N1 (n=4) were often in agreement, followed by N2b (n=1) (Figure 7).

**Figure 8:**
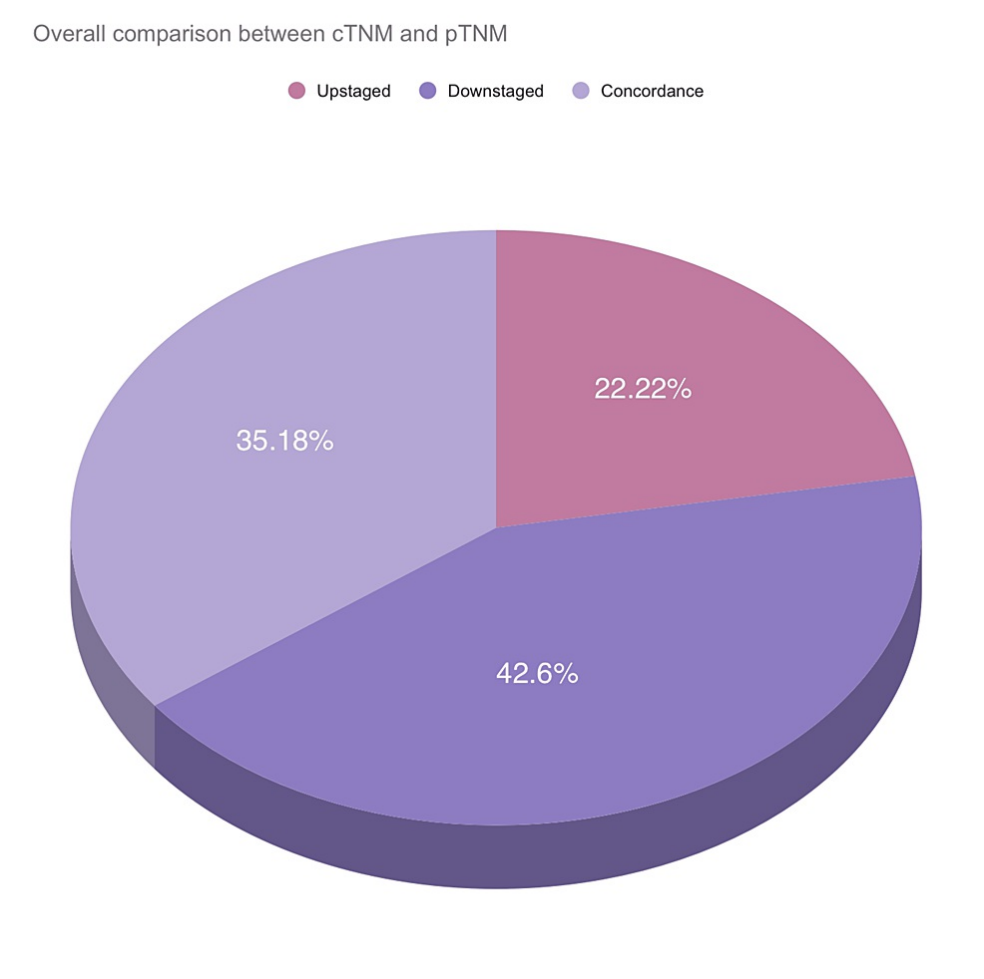
Overall comparison between clinical tumor staging and pathological tumor staging among the included cases

Statistical analysis revealed significant differences between the clinical and pathological nodal status with a p-value of 0.002 (Table 1). With the total comparison between pathological and clinical tumor staging, 42.60% were downstaged, 35.18% were concordant and 22.22% were upstaged (Figure 8).

**Figure 9:**
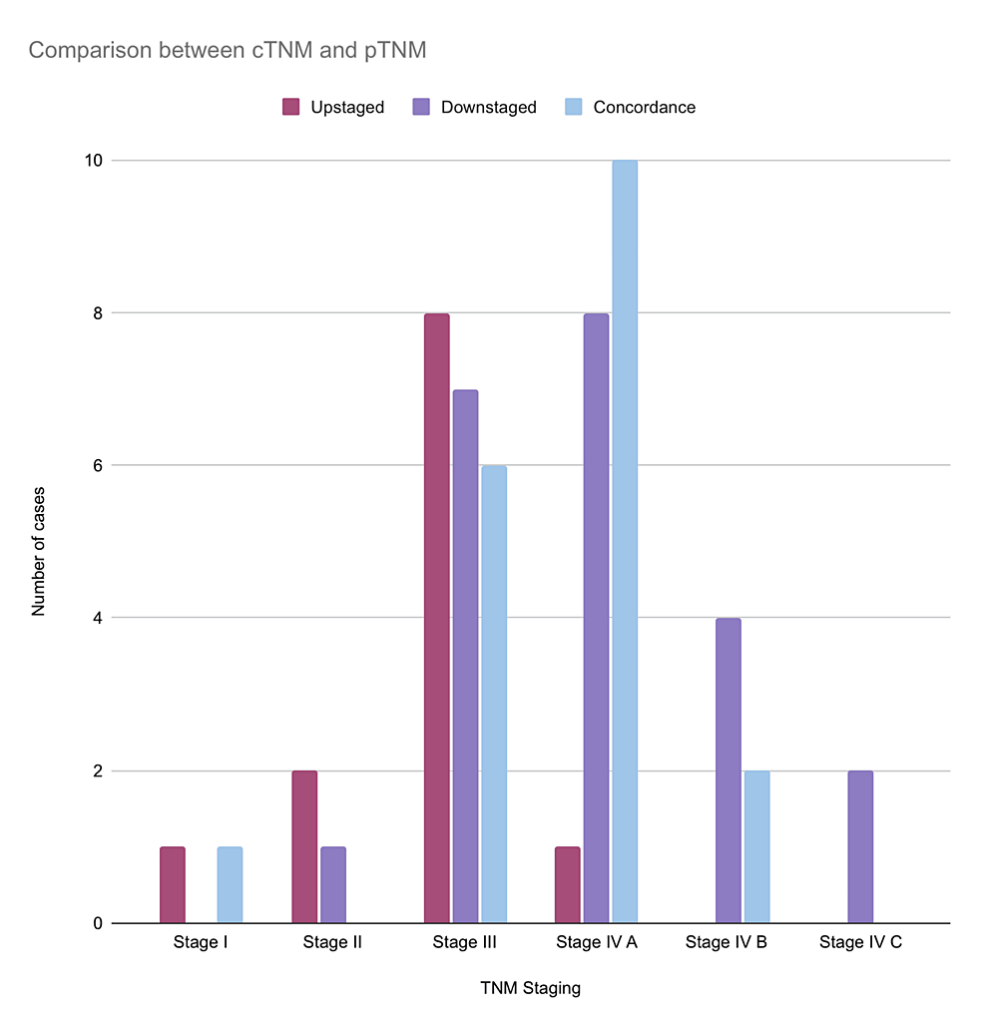
Comparison between clinical TNM staging and pathological TNM staging among the included cases T: primary tumor size; N: regional lymph node; M: distant metastasis

In particular, Stage III (n=8) was often upstaged, followed by Stage II (n=2), Stage I (n=1), and Stage IVA (n=1). Stage IVA (n=8) was frequently both downstaged and in concordance, followed by Stage III (n=7) and Stage IVB (n=4) (Figure 9).

**Figure 10:**
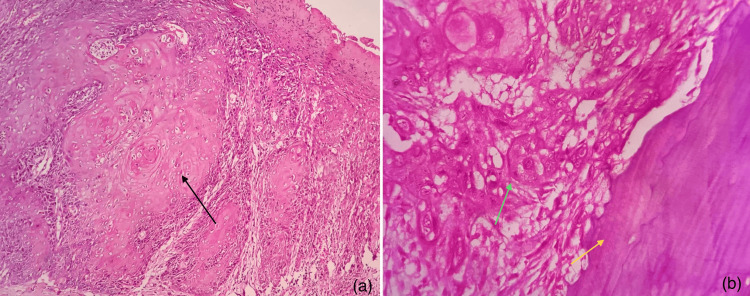
Photomicrographs of oral squamous cell carcinoma depicting pathological tumor criteria (H&E) (a) pT1 showing malignant epithelial cells in the superficial connective tissue stroma without any invasion to deeper structures (H&E, 10x) - black arrow depicting keratin pearl formation. (b) pT4a with malignant epithelial cells (green arrow) invading the adjacent cortical bone (yellow arrow) (H&E, 40x).

Figure 10 shows the photomicrographs of pathological tumors included in our study. pT1 was squamous cell carcinoma within the superficial connective tissue stroma and pT4a showed invasion of malignant epithelial cells into the cortical bone.

ANOVA test was performed for tumor size of clinical and pathological criteria. It was not significant with a p-value of 0.080 (Table 2). ANOVA comparison of nodal status among the study group did not attain statistical significance (Table 2).

Hence, Tukey's HSD posthoc tests were performed. Considering tumor size between the various subgroups, T1, T2, T3, T4a, and T4b did not reveal any statistical significance (Table 3).

**Table 3:** Tukey's HSD posthoc test for tumor size HSD: Honest Significant Difference

(I) cT	(J) cT	Mean Difference (I-J)	Std. Error	Sig.
					T1	T2	0.17949	0.37784	0.989
T3	0.17949	0.37784	0.989		
T4a	0.83333	0.36648	0.171		
T4b	0.33333	0.44200	0.942		
T2	T1	-0.17949	0.37784	0.989
	T3	0.00000	0.30028	1.000
	T4a	0.65385	0.28586	0.166
	T4b	0.15385	0.37784	0.994
T3	T1	-0.17949	0.37784	0.989
	T2	0.00000	0.30028	1.000
	T4a	0.65385	0.28586	0.166
	T4b	0.15385	0.37784	0.994
T4a	T1	-0.83333	0.36648	0.171
	T2	-0.65385	0.28586	0.166
	T3	-0.65385	0.28586	0.166
	T4b	-0.50000	0.36648	0.653
T4b	T1	-0.33333	0.44200	0.942
	T2	-0.15385	0.37784	0.994
	T3	-0.15385	0.37784	0.994
	T4a	0.50000	0.36648	0.653

Tukey's HSD posthoc tests for nodal involvement between the various subgroups - Nx, N0, N1, N2a, N2b, and N2c - were statistically significant only for N0 vs N1 (Table 4).

**Table 4:** Tukey HSD posthoc test for nodal involvement Only the comparison of N0 vs N1 had statistical significance (0.034*). All the other comparisons were not significant. HSD: Honest Significant Difference

(I) cN	(J) cN	Mean Difference (I-J)	Std. Error	Sig.
					N0	N1	-0.73737^*^	0.24634	0.034*
N2a	-0.55556	0.43672	0.709		
N2b	-0.22222	0.43672	0.986		
N2c	-0.75556	0.36538	0.251		
N1	N0	0.73737^*^	0.24634	0.034*
	N2a	0.18182	0.39503	0.990
	N2b	0.51515	0.39503	0.690
	N2c	-0.01818	0.31437	1.000
N2a	N0	0.55556	0.43672	0.709
	N1	-0.18182	0.39503	0.990
	N2b	0.33333	0.53487	0.971
	N2c	-0.20000	0.47840	0.993
N2b	N0	0.22222	0.43672	0.986
	N1	-0.51515	0.39503	0.690
	N2a	-0.33333	0.53487	0.971
	N2c	-0.53333	0.47840	0.798
N2c	N0	0.75556	0.36538	0.251
	N1	0.01818	0.31437	1.000
	N2a	0.20000	0.47840	0.993
	N2b	0.53333	0.47840	0.798

Clinical assessment using a full body scan revealed two positive cases of distant metastasis. As a biopsy was not performed to confirm the distant metastasis histopathologically, distant metastasis (M) was not included in the comparison.

## Discussion

Despite improved treatment modalities, clinicopathological prognostic factors remain insufficient for predicting the recurrence and survival of OSCC. Hence, the survival of patients has remained unchanged over the last few decades. OSCC tumor staging and histopathological tumor grading are imperative for management. They impact risk assessment and form the basis for determining individualized treatment options. Additionally, OSCC is known to present with verifiable histological behavior patterns [10]. When the histological staging was first developed in the early 1900s, cancer was classified as a local, regional, or distant disease. French scientist Pierre Denoix developed this idea between 1943 and 1952, establishing the basis for the current TNM (tumor, node, metastasis) approach by categorizing malignancies according to their anatomical location and spread. This system was adopted in 1953 by the Union for International Cancer Control (UICC) (Europe) whereas the AJCC in 1959 modified the system. As both methods became widely used, the AJCC and UICC TNM committees worked together to develop a single system in 1982 [10-12].

The best possible management of head and neck cancer has been greatly aided by the staging system of AJCC/UICC that followed guidelines and recommendations from organizations like the US NCCN (National Comprehensive Cancer Network). However, as knowledge advances, current classifications become old and require adjustment and improvement. In particular due to an increase in Human Papilloma Virus (HPV)-associated oropharyngeal squamous cell carcinoma (OPSCC) [13,14]. The AJCC recently issued a manual for staging oral cancer (eighth edition) in 2018. Rajapakshe et al. [15] and Geum et al. [16] reported that the TNM stage was found to have a significant influence on the prognosis of OSCC patients (p<0.001). Furthermore, this study is vital for evaluating and validating the predictive efficacy of tumor staging, a critical clinical prognostic factor in the Indian population.

According to data from 970 patients, the floor of the mouth, soft palate, gingiva, buccal mucosa, and hard palate were the sites with the highest prevalence of OSCC, accounting for around 50% of all the cases [17]. Taiwan's Ministry of Health and Welfare's Health Promotion Administration stated that oral cavity cancer most frequently affects the buccal mucosa followed by the tongue [18]. According to our research, the most common site observed was the alveolar mucosa, most likely as a result of the common usage of betel quid and gutkha in this region. The location and the placement of betel quid is crucial where this cancer primarily develops. Moreover, chewing betel quid (with or without tobacco) and other smokeless tobacco products, which are popular in South Asia, are closely associated with the development of OSCC. This suggested a particular preference for placement contributes to OSCC in the alveolar mucosa. Shah and Gil observed that the biological characteristics of OSCC varied based on the main site [19]. Carcinomas on the mucosal lip showed a good prognosis, whereas carcinomas on the floor of the mouth, the anterior two-thirds of the tongue, and the lower alveolar ridge showed comparatively worse prognoses and a significant probability of spreading to adjacent lymph nodes [20]. Our analysis usually observed tumors classified as T3 or T4, which is in line with findings in the literature where T3 is frequently reported. However, N0 was the most generally reported, followed by N2b. On the other hand, pathological N2 and N3 nodal involvement were frequently reported in the previous literature.

When compared to pathological tumor staging, T2 tumors and T4a tumors found during clinical staging were frequently upstaged and downstaged, respectively. This disparity could be due to the radiographic evidence of bone erosion which clinicians mistook as evidence of bone involvement. On radiographs, however, the clinical T stage of T4a matched the pathological T staging when there were evident signs of bone involvement, such as a fracture or perforation. Hence, precise radiographic examination plays a vital role in predicting bone status clinically. Various imaging modalities are investigated in several prospective and retrospective investigations to assess mandibular invasion in OSCCs. Among these are the orthopantomogram (OPG), bone scan, magnetic resonance imaging (MRI), CT, Denta scan, and single photon emission computed tomography (SPECT). These investigations contrasted periosteal stripping and clinical assessment. Although initial evaluations concluded that clinical examination was more reliable than imaging, further research unequivocally showed that imaging is crucial for determining mandibular invasion. SPECT and MRI revealed the highest sensitivity, ranging from 96% to 97%, whereas CT scans provided the highest specificity, at 87% [21-23]. MRI is also useful for depicting the T stage, another important aspect affecting treatment and prognosis. OPG's drawbacks include its inability to detect soft tissue, the area about symphysis menti, and its inability to identify bone erosion until 30-75% of the mineral has been lost [24].

Since the primary technique for determining the clinical nodal status was lymph node palpation, N1 staging was often both upstaged and downstaged. Inflammation can also cause palpable lymph nodes, which could be mistaken for positive lymph nodes. This is important because precise confirmation avoids unnecessary dissection of lymph nodes. Other characteristics, such as extranodal extension and the size of deeply placed lymph nodes, which can only be evaluated histopathologically, may also play a role in the frequent upstaging of N1 staging. Research has shown that sentinel lymph node biopsy is advised instead of elective neck dissection for about 70-80% of patients with cN0 oral cancer [25,26]. This emphasized how important it is to forecast preoperative staging accurately, especially with the accuracy of preoperative cervical lymph node staging. In many medical institutes, ultrasonography, MRI, and contrast-enhanced CT are frequently used for identifying local metastatic lymph nodes. It has been found that imaging works better than the clinical cervical palpation method [27]. Nevertheless, up to 20-46% of cN0 oral carcinomas had occult metastases, which were not visible clinically or radiologically [28]. However, there is disagreement over the best imaging method since earlier research has demonstrated comparable diagnostic precision in identifying nodal metastases [29,30]. Therefore, a major factor in deciding the course of treatment that patients get is the preoperative clinical and radiological technique used.

There are limitations in our study as it was obtained from a single institution. We did not include patients who underwent alternate forms of therapy for OSCC. We did not correlate the quality of life outcomes with clinical and pathological TNM staging of OSCC. We have not compared the deleterious habit-related OSCC with non-habit OSCC cases. We also did not evaluate the role of HPV in our study.

## Conclusions

The differences observed in this study's preoperative clinical staging and postoperative pathological staging underscore the need for accurate clinical and radiological patient evaluations for accurate diagnosis. Treatment decisions are greatly influenced by the diagnosis. However, nodal status assessments by clinical palpation and prediction of tumor extension using radiographs are not always precise. Additionally, because of their complexity and expenses, CT and MRI are not usually performed for individuals with early stages of OSCC. Thus, choosing the optimal surgical approach requires careful consideration of the preoperative examination technique for each patient to improve overall survival and quality of life.
